# Effect of the order of concurrent training combined with resistance and high‐intensity interval exercise on mTOR signaling and glycolytic metabolism in mouse skeletal muscle

**DOI:** 10.14814/phy2.14770

**Published:** 2021-03-02

**Authors:** Takanaga Shirai, Hideto Hanakita, Kazuki Uemichi, Tohru Takemasa

**Affiliations:** ^1^ Graduate School of Comprehensive Human Sciences University of Tsukuba Tsukuba Ibaraki Japan; ^2^ Research Fellow of Japan Society for the Promotion of Science Chiyoda‐ku Tokyo Japan; ^3^ Faculty of Health and Sport Sciences University of Tsukuba Tsukuba Ibaraki Japan

**Keywords:** concurrent training, glycolysis, high‐intensity interval exercise, mTOR signaling, resistance exercise

## Abstract

Athletes train to improve strength and endurance to demonstrate maximum performance during competitions. Training methods vary but most focus on strength, endurance, or both. Concurrent training is a combination of two different modes of training. In this study, we combined resistance exercise (RE) and high‐intensity interval exercise (HIIE) to investigate the influence of the order of the concurrent training on signal molecules on hypertrophy and glycolysis in the skeletal muscle. The phosphorylation levels of mechanistic target of rapamycin (mTOR) signals, p70 S6 kinase (p70S6 K), ribosomal protein S6 (S6), and glycogen synthase kinase beta (GSK‐3β) were significantly increased in the HIIE first group compared with the control group. The combined training course did not affect the glycogen content and expression levels of proteins concerning glycolytic and metabolic capacity, suggesting that a combination of HIIE and RE on the same day, with HIIE prior to RE, improves hypertrophy response and glycolysis enhancement.

## Graphical abstract

In this study, we performed concurrent training combining RE and HIIE and examined the expression dynamics of mTOR signaling and signaling molecules of glycolytic metabolism. As a result, it was suggested that the expression of the signaling molecules was enhanced by resistance exercise first in order to increase the effect of both exercises.

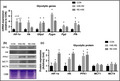

## 1 INTRODUCTION

Skeletal muscle quality and quantity are closely related to the competitive performance of athletes, who (especially ball game players) require both muscle strength and endurance abilities, so they take combination for getting both of them. Concurrent training (CT) is a combination of two modes of exercise. By the CT with resistance exercise and endurance exercise, it has been reported to reduce maximum muscle strength and muscle hypertrophy efficiency compared to RE alone, which are called the interference effect (Hickson, 1980). Recently, we investigated the effect of the order of CT, which combines resistance and endurance exercises, on muscle hypertrophy signaling and reported that resistance exercise first is more likely to prevent interference effects in mouse skeletal muscle. The amount, time, and intensity of training influence the training outcomes. Among these, it is reported that the interference effect can be evoked by exercise time and intensity of endurance exercise (Fyfe et al., 2016). Therefore, we focused on high‐intensity interval exercise (HIIE). HIIE is a training method in which all‐out or all‐out equivalent high‐intensity exercise is performed for several seconds to several minutes, and several sets are repeated with a short rest interval in between sets. This efficiency improves the glycolytic enzyme activity in a short time compared with traditional endurance exercise and causes adaptation to increase the production of ATP (Gibala, 2009). In recent reports, HIIE positively improved maximal oxygen uptake and metabolic markers compared with endurance training (Tjønna et al., 2008). HIIE has several benefits such as improved aerobic and glycolytic metabolism and increased muscle strength especially in masters athletes (Herbert et al., 2017; Tabata et al., 1996).

Recent studies have demonstrated the ability of HIIE to alter molecular signals involved in oxidative metabolism. First of all, HIIE activates peroxisome proliferator‐activated receptor γ coactivator‐1α (PGC‐1α), which is the master regulator gene of oxidative metabolism, and increases mitochondrial ATP production (Gibala, 2009). Furthermore, HIIE produces energy through glycolytic metabolism, thereby increasing the levels of glycolytic enzymes such as phosphofructokinase (Pfk), lactate dehydrogenase (Ldh), and glycogen synthase (Gys) in the skeletal muscle (Jacobs et al., 1987; Linossier et al., 1997) as well as the hypoxia‐inducible factor (HIF‐1α) (Abe et al., 2015), which was initially identified as an important protein for hypoxic adaptation and reported to be involved in the adaptation of endurance exercises such as capillary neovascularization (Semenza & Wang, 1992).

The expression level of Hif‐1α increases by not only HIIE but also RE (Ameln et al., 2005). Hif‐1α in skeletal muscle fibers is more highly expressed in fast type than in slow type (Pisani & Dechesne, 2005), and it has been clarified that upregulation of Hif‐1α can hasten the expression system from slow fiber to fast (Lunde et al., 2011). Therefore, in addition to increasing oxidative metabolic and glycolytic capacity, High Intensity Interval Training (HIIT) may enhance HIF‐1α more effectively than RE.

For the reasons stated above, if CT combined with HIIE can be performed, it may be more time‐efficient than CT combined with endurance exercise to enhance the effect of exercise. Previous CT studies have examined the effects of a combination of long‐term endurance and RE with relatively low intensity (Hickson, 1980). In this study, to examine the effects of CT combined with HIIE and RE in skeletal muscle, we focused on signal molecules concerning muscle hypertrophy, glycolysis, and oxidative metabolism signals. We hypothesized that CT combined with resistance exercise and HIIE could activate hypertrophic and glycolytic metabolic signals regardless of the order.

## 2 MATERIAL AND METHODS

### 2.1 Animals

All experimental procedures performed in this study were approved by the Institutional Animal Experiment Committee of the University of Tsukuba. Male mice aged 7–8 weeks from the Institute of Cancer Research (ICR) (Tokyo Laboratory Animals Science Co.) were housed in holding facilities under controlled conditions: temperature, 22°C ± 2°C; humidity, 55% ± 5%; and lighting, light/dark cycle for 12 hours; the mice were given ad libitum access to food and water. Upon completion of experimental treatments, the mice were killed by cervical dislocation, and their lower limb muscles were then dissected 24 hr after the last exercise session, weighed quickly frozen in liquid nitrogen, and stored at −80°C until analysis.

### 2.2 Training methods

#### 2.2.1 Resistance exercise protocol

The resistance exercise protocol was carried out as previously described (Ogasawara et al., 2016; Shirai et al., 2020). Briefly, under inhaling isoflurane anesthesia (2%, KN‐1701; Natsume), both lower limbs of each mouse were shaved and cleaned with alcohol wipes. The mice were positioned with their foot on a footplate (with an ankle joint angle of 90°) in the prone position. The triceps and the calf muscle were stimulated percutaneously with electrodes connected to an electric stimulator and isolator (Ag/AgCl, Vitrode V; Nihon Kohden). The gastrocnemius muscle was isometrically exercised (stimulation for 3 s, 10 contractions, with intervals of 7 s between contractions; total of five sets with 3‐min intervals between sets). The voltage (30 V) and stimulation frequency (100 Hz) were adjusted to produce maximal isometric tension. This exercise protocol is known to increase the anabolic signaling activity (Ogasawara et al., 2016) and induces significant muscle hypertrophy, simulating long‐term training (Ogasawara et al., 2013).

#### 2.2.2 High‐intensity interval training protocol

The HIIE protocol was carried out as previously described (Abe et al., 2015). A barrel filled with water to a depth of 60 cm was used as a swimming pool, and the water temperature was maintained 37℃ during the exercise. The mice swam for 20 s for a maximum of 10 sets or until exhaustion, with weights equivalent to 10% of their body weight tied at the proximal ends of their tails. After each set of swimming exercises, the mice were retrieved from the water and allowed to rest for 10 s. Immediately after HIIE was finished, all mice were wiped dry with a Kim towel to dry their hair.

### 2.3 Combined training

We evaluated the order of CT. The animals were randomly assigned to either HIIE exercise before the resistance exercise group (HIIE‐RE) or HIIE after the resistance exercise group (RE‐HIIE). A 1‐hr rest period was provided between exercises. The animals were trained thrice a week for 3 weeks. (18 exercise sessions in total). In addition, the non‐exercise control (CON) group eliminates the effects of the environment without electrical stimulation or without water in a barrel (CON group anesthetized nine times in total). Non‐exercise control (CON) animals were euthanized at the basal state.

### 2.4 Western blotting

Excised gastrocnemius muscles were immediately frozen in liquid nitrogen, and total muscle protein was extracted with lysis buffer containing 50 mM HEPES (pH: 7.6), 150 mM NaCl, 10 mM EDTA, 10 mM Na_4_P_2_O_7_, 10 mM NaF, 2 mM Na_3_VO_4_, 1% (vol/vol) NP‐40, 1% (vol/vol) Na‐deoxycholate, 0.2% (wt/vol) SDS, and 1% (vol/vol) complete protease inhibitor cocktail (Nacalai Tesque Inc.). Protein concentrations were measured using a Protein Assay Bicinchoninate Kit (Nacalai Tesque Inc.). Before SDS‐PAGE, an aliquot of the extracted protein solution was mixed with equal volume of sample loading buffer containing 1% (vol/vol) 2‐mercaptoethanol, 4% (wt/vol) SDS, 125 mM of Tris‐HCl (pH: 6.8), 10% (wt/vol) sucrose, and 0.01% (wt/vol) bromophenol blue. The mixture was then heated at 97°C for 3 min. Ten micrograms of protein was separated on an SDS‐polyacrylamide gel and electrically transferred to an ImmunoBlot PVDF membrane (Bio‐Rad Laboratories). The blot was blocked by Blocking One (Nakalai Tesque Inc.) for 1 h at room temperature and incubated with primary antibodies overnight at 4℃ in TBS containing 0.1% Tween 20. Signals were detected using the Immunostar Zeta or LD (Wako Chemicals), quantified by C‐Digit (LI‐COR Biosciences), and expressed as arbitrary units. The expression levels of each protein were normalized to those of glyceraldehyde 3‐phosphate dehydrogenase.

#### 2.4.1 Primary antibodies for western blotting

The following primary antibodies were used for western blotting: protein kinase B (Akt) (9272; Cell Signaling Technology), p‐Akt (#4060S; Cell Signaling Technology), mechanistic target of rapamycin (mTOR) (#2983; Cell Signaling Technology), p‐mTOR (#2971; Cell Signaling Technology), p70 S6 kinase (p70S6 K) (#9202; Cell Signaling Technology), p‐p70S6 K (#9205; Cell Signaling Technology), 4E‐binding protein 1 (4E‐BP1) (#9452; Cell Signaling Technology), p‐4E‐BP1 (#9459; Cell Signaling Technology), S6 ribosomal protein (S6) (#2217; Cell Signaling Technology), p‐S6 (#4858S; Cell Signaling Technology), glycogen synthase kinase 3β (GSK3β) (#12456; Cell Signaling Technology, Danvers, MA, USA),p‐GSK3β (#5558; Cell Signaling Technology), hypoxia inducible factor 1‐α (HIF‐1α) (NB100‐105; Novus Biologicals), hexokinase (HK) (sc‐374091; Santa Cruz), phosphofructkinase (PFK) (sc‐97025, Santa Cruz), monocarboxylate transporter 1 (MCT1) (QUIAGEN), MCT4 (Quiagen), and oxidative phosphorylation (OXPHOS) (ab110413; Abcam).

### 2.5 Glycogen measurement

Muscle glycogen content was determined, as described by Hassid and Abraham (8). In brief, 100 mg of gastrocnemius muscle was digested in 300 µl of 30% KOH for 30 min at 100°C. Saturated sodium sulfate (50 µl) was added, and glycogen was precipitated by adding 500 µl of 95% ethanol. The solution was heated to 100°C, stirred, then cooled, and centrifuged at 1,600 *g*. The supernatant was decanted, and the remaining alcohol was removed by heating. The pellet was dissolved in 200 µl of H_2_O and precipitated with 250 µl of 95% ethanol. After centrifugation at 1,600 *g*, the supernatant was decanted, and the remaining alcohol was removed by heating. Purified glycogen was hydrolyzed in 600 µl of 0.6 N HCl at 100°C for 2.5 h. The solution was cooled, and the glucose concentration was measured using the Glucose CII test kit. For calculating the amount of glycogen from the concentration of glucose in the hydrolyzed glycogen sample, a conversion factor of 0.93 was used.

### 2.6 RNA isolation and real‐time polymerase chain reaction (PCR)

Total RNA was isolated from frozen whole gastrocnemius muscles using the Trizol reagent (Invitrogen). The quantity and quality of RNA were validated with Nanodrop (Thermo Fisher Scientific). Complementary DNA was synthesized using the PrimeScript RT Master Mix (Takara Bio, Inc.). qRT‐PCR was performed with the Thermal Cycler Dice Real‐Time System using SYBR Premix Ex taq II (Takara Bio, Inc.). The PCR protocol was as follows: denaturation for 15 s at 95°C, then annealing, and extension for 40 seconds at 60°C (40 cycles). The dissociation curve for each sample was analyzed to verify the specificity of each reaction. The relative mRNA expression levels of the target genes were determined by the delta‐delta Ct method and normalized to the expression of TATAbox binding protein (Tbp). The primer sequences are shown in Table 1.

**TABLE 1:** Primer sequence for RT‐PCR

Gene	Forward primer (5'−3’)	Reverse primer (5'−3’)
*Hif−1α*	GGCAGCGATGACACAGAAAC	AGGTAAAGGAGACATTGCCAGG
*Hk*	GGCTAGGAGCTACCACACAC	AACTCGCCATGTTCTGTCCC
*G6pd*	CAGCTCAGTCAAAGCACACG	ATGTTTTCTGAGTTTAGTTGCCGC
*Pygm*	GTACAAGAACCCAAGAGAGTGGA	CGAGAAGGTTCAACACCCCA
*Pgd*	TCCGTAAGGCCCTCTATGCT	TTGAGGGTCCAGCCAAACTC
*Pfk*	ACAATCTGCAAGAAAGCAGCG	TGCAGCAAATCGCTTGGG
*Pgc‐1α*	TTCCACCAAGAGCAAGTAT	CGCTGTCCCATGAGGTATT
*CytoC*	CACGCTTTACCCTTCG	CTCATTTCCCTGCCAT
*Cox1*	CTAGCCGCAGGCATTACTAT	TGCCCAAAGAATCAGAACAG
*Nd4*	ATTATTATTACCCGAT	ATTAAGATGAGGGCAA
*Tbp*	CTGCCACACCAGCTTCTGA	TGCAGCAAATCGCTTGGG

### 2.7 Statistical analysis

Data were analyzed with the Graph Pad 7.0 software, and the values are reported as the means ± standard error (SE). All data analyzed with one‐way analysis of variance (ANOVA). When a significant P value was obtained, the statistical significance (post hoc test) was calculated according to Tukey's methods. The statistical significance was defined as *p* < 0.05.

## 3 RESULTS

### 3.1 Body and muscle weight

**FIGURE 1:**
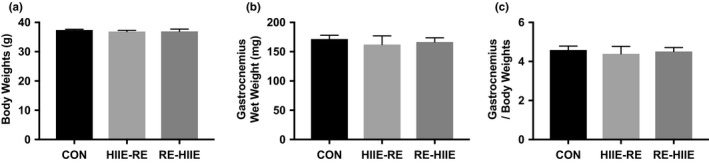
Animal characteristics. Values are mean ± SEM (n = 5 per group)

The characteristics of each group of mice are presented in Figure 1. First, we measured the body weight and gastrocnemius wet weight for each mouse throughout the exercise period of 3 weeks. Body weight and gastrocnemius wet weight were not altered in all groups (Figure 1). Three weeks’ combined resistance and high intensity interval training were not altered body compositions.

### 3.2 Glycogen content

**FIGURE 2:**
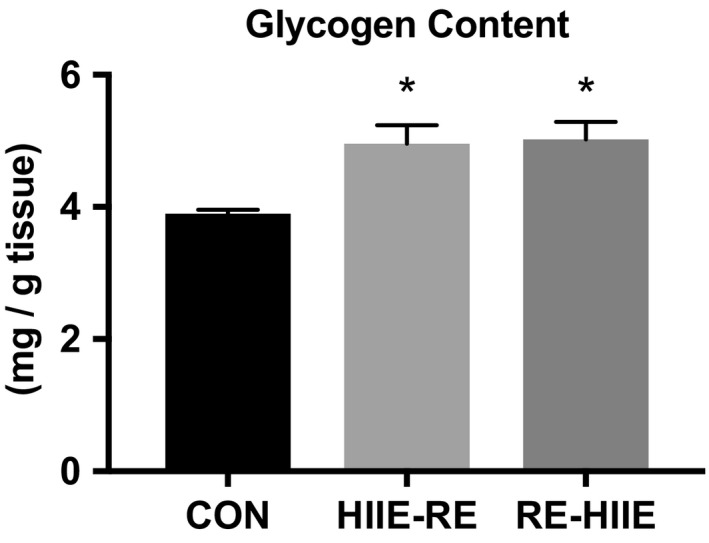
Glycogen content after combined resistance and high‐intensity interval training in mouse skeletal muscle. Data are presented as mean +SEM. n = 5 each group. *p < 0.05 vs CON group

Next, we measured the glycogen content of each group, as an effect of combined training. Glycogen is an essential energy source during HIIE or RE, and its content represents an important storage form of energy for anaerobic metabolism. The glycogen content was significantly increased in the HIIE‐RE and RE‐HIIE group but not different by the order (Figure 2).

### 3.3 mTOR signaling molecules

**FIGURE 3:**
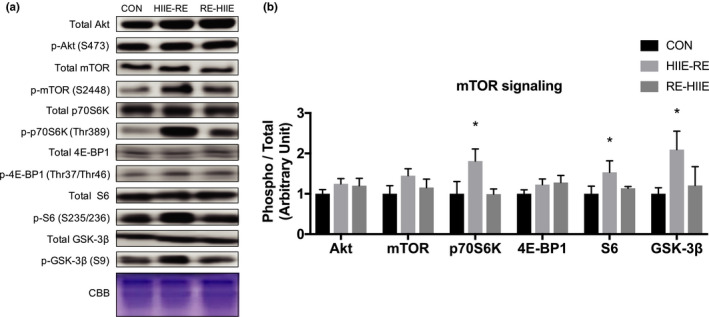
Effect of the order of combined resistance and high‐intensity interval training on mTOR signaling in mouse skeletal muscle. (a) Immunoblots patterns and (b) protein expression levels of mTOR signaling. Data are presented as mean +SEM. n = 5 each group. *p < 0.05 vs CON group

To investigate the effects of order of combined RE and HIIE, we measured protein expression levels of the mTOR signal pathway. The expression of proteins associated with mTOR signaling (p70S6 K, S6, GSK‐3β) was significantly increased in the HIIE‐RE group compared with the CON group, but other proteins were not significantly different (Figure 3).

### 3.4 Molecules concerning glycolysis

**FIGURE 4:**
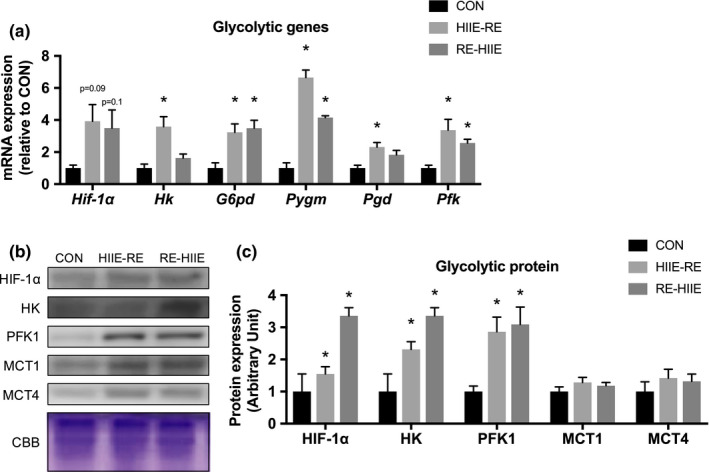
Effect of the order of combined resistance and high‐intensity interval training on glycolytic genes and proteins in mouse skeletal muscle. (a) mRNA expression levels of glycolytic genes, (b) Immunoblots patterns and (c) protein expression levels of glycolytic proteins. Data are presented as mean +SEM. n = 5 each group. *p < 0.05 vs CON group

Next, we measured the expression of genes and proteins associated with glycolysis, which is the main effect of HIIE. The expression levels of genes for *Hk*, *Pgd*, and *Pfk* were significantly increased in the HIIE‐RE group compared with the CON group. In addition, the gene expression levels of *G6pd*, *Pygm*, and *Pfk* were significantly increased in both HIIE‐RE and RE‐HIIE groups compared with the CON group. Next, the expression levels of proteins for HIF‐1α, HK, and PFK1 were significantly increased in both HIIE‐RE and RE‐HIIE groups compared with the CON group. In addition, the expression levels of MCT1 and MCT4, which are involved in lactate transport, did not changed between groups. Therefore, there was also no difference by the order of combined resistance and high intensity interval training on glycolysis (Figure 4).

### 3.5 Molecules concerning aerobic metabolism

**FIGURE 5:**
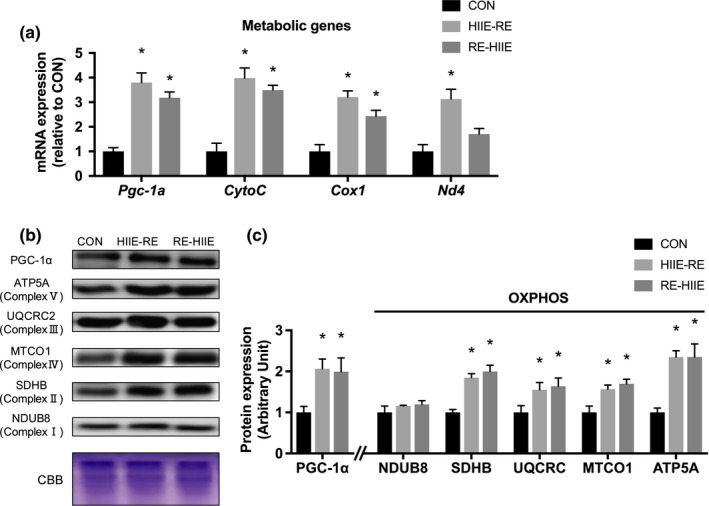
Effect of the order of combined resistance and high‐intensity interval training on mitochondrial genes and complex chains in mouse skeletal muscle. (a) mRNA expression levels of mitochondrial genes, (b) immunoblots patterns, and (c) protein expression levels of PGC‐1α and OXPHOS proteins. Data are presented as mean +SEM. n = 5 each group. *p < 0.05 vs CON group

Despite the unaltered body weight and gastrocnemius muscle, 3 weeks’ combined RE and HIIE significantly increased the gene expression level of *Pgc*‐*1α*, known as the master regulator of mitochondrial biogenesis, in both HIIE‐RE and RE‐HIIE groups. The gene expression levels of mitochondrial proteins (*CytoC*, *Cox1*, *Nd4*), significantly increased after training, but the difference by the order was not demonstrated (Figure 5). Then, we measured members of respiratory chain complex related to oxidation phosphorylation (OXPHOS). The protein levels of SDHB, UQCRC, MTCO1, and ATP4A were significantly increased in both HIIE‐RE and RE‐HIIE groups compared with the CON group, but no significant difference was observed in terms of the order of combined RE and HIIE on mitochondrial biogenesis.

## 4 DISCUSSION

In this study, we investigated the difference by the order of CT combined with RE and HIIE, focusing on the molecular responses in mice skeletal muscle. As a result, the expression levels of proteins related to the mTOR signal were upregulated by performing HIIE first, and the responses of molecular signals involved in glycolysis/oxidative metabolism were not changed by the order. Most previous studies focused on changes in the expression levels of signal molecules for each exercise alone, and there were few reports that analyzed signal molecules in combined two types of exercise modes, RE and HIIE. In this study, we focused on the order of combined training with RE and HIIE and observed molecular signals related to mTOR, glycolysis, and oxidative metabolism. Here, we could propose a CT protocol which is suitable for athletes who require complicated energy mechanisms such as the ball game players.

The CT protocol applied in this study (3 times a week for 3 weeks) did not affect the body weight and muscle wet weight. The mice were performed exercises twice (RE +HIIE) in a day, so we think exercise volumes were sufficient in this study, but the experiment lasted for 3 weeks, which might not affect mice body weight or muscle wet weight. HIIT with running for 4 weeks reduced body weight but did not change the weight of gastrocnemius muscles in rat and mouse (Hoshino et al., ,,^2013^, ^2015^). On the other hand, 8 weeks of HIIT with running increased muscle weight and CSA compared to 4 weeks of HIIT with running (Goh et al., 2019). In order to ascertain changes in muscle wet weight or cross‐sectional area, it may be possible to prolong the experimental period.

We measured the muscle glycogen content after 3 weeks training, which increased by CT, but without difference by the order. In previous studies using animal models, it has been reported that RE using electrical stimulation reduces the muscle glycogen content soon after exercise (Ogasawara et al., 2017). Another animal research was reported that HIIE for 6 weeks increased the muscle glycogen content (Abe et al., 2015) and that muscle glycogen was used as a quick energy source and the muscle glycogen content was increased as adaptation for HIIE. Studies performed on human subjects clarified that combined RE and HIIE decreased muscle glycogen content in the CT group compared with RE alone (Fyfe et al., 2016) and suggested that the combination of RE and HIIE increased the utilization of muscle glycogen. In our study, no difference was observed in the muscle glycogen content depending on the order of CT, presumably because the exercise volume in both groups was the same.

We measured phosphorylation levels of p70S6 K, S6, and GSK‐3β, which are the proteins of the mTOR signaling pathway (Bodine et al., 2001; Miyazaki & Esser, 2009), and found they were increased by CT performed with HIIE first. In a previous study using rats, 8‐week sprint interval treadmill running was reported to cause muscle hypertrophy via the IGF‐1/Akt/FoxO and myostatin/Smad signals (Biglari et al., 2020). Since no significant difference was demonstrated in the expression levels of mTOR and p70S6 K in the previous study, it is considered that the mTOR signal was activated mainly by RE (Brook et al., 2015). CT combining RE and HIIE in humans activated the proteins of mTOR signal in human subjects (Fyfe et al., 2016). The activation levels of mTOR signals were different depending on the order of protocol; thus, demonstrating the effect of RE was promoted by performing HIIE first. The exercise effects of HIIE include a decrease in muscle glycogen immediately after exercise (Takeda & Takemasa, 2015) and an increase in blood lactate level due to an increase in glycogen utilization as an energy substrate (Abe et al., 2015), an increase in expression of angiogenic master genes such as Hif‐1α by HIIE, and intramuscular and blood acidification (Bishop et al., 2004; Hollidge‐Horvat et al., 2000). Among these responses, elevated blood lactate has attracted attention as a trigger to increase the mTOR signal (Cerda‐Kohler et al., 2018). Future studies need to examine whether exercise induced increases in blood lactate levels affects mTOR signal responses.

In the RE‐HIIE group that underwent the resistance exercise first, the mTOR signal did not activate. In a previous study, 80% VO_2_ HIIE on a treadmill reduces atrogin‐1, MAFbx gene expression downstream of the IGF‐1/Akt/FoxO cascade, but does not alter mTOR signaling (Cui et al., 2019). In this study, we used the swimming exercise model, which is considered not to enhance the expression of mTOR signal. In the swimming exercise, water temperature also affects the mTOR signal (Fyfe et al., 2019) (Ihsan et al., 2020), which thus requires consideration of the mode of exercise (swimming or running), as this could produce different results. Furthermore, it has been reported that the molecular response of exercise mainly depends on the exercise mode that was applied last in CT (Ogasawara et al., 2014). In that report, mTORC1 signaling induced by a prior bout of RE was downregulated by a subsequent bout of EE (Ogasawara et al., 2014), and similar results were demonstrated in this study using HIIE.

The order of RE and HIIE did not affect glycolytic metabolism. The level protein expression of HIF‐1α, the master gene of glycolytic metabolism, was elevated in both CT groups. In recent studies, it has been reported that HIF‐1α is increased not only by HIIE but also by RE (Ameln et al., 2005; Lunde et al., 2011), and the same exercise effect was obtained in our CT protocol. In a study that followed the temporal changes in the Hif‐1α gene expression, it peaked 3 hours after exercise, in the condition that the total exercise time per session of the experimental protocol was less than 1 h and 30 min (RE 8 min 20 s, 1 h rest between sessions, HIIE 5 min), although their protocol did not affect the expression level of HIF‐1α significantly. It has been reported that PFK1 and HK2 are important enzymes for glucose metabolism (Linossier et al., 1997), and CT shows high levels of PFK1 and HK2 proteins in this study as well. Subsequently, we measured genes related to glycolytic metabolism. The results showed that *Hk* and *Pgd* were significantly increased in the HIIE‐RE group compared with the CON group. HK is one of the most important enzymes involved in skeletal muscle glucose metabolism (Meijer, 1991; Wagner et al., 1978). In skeletal muscle, muscle‐specific Akt1 plays a critical role in mediating resistance training‐induced muscle hypertrophic growth and protein synthesis. Akt1 overexpression mice increase pentose phosphate pathway metabolites and enzymes, such as ribose‐5‐phosphate, G6PD, and PGD (Wu et al., 2017). In the present study, the gene expression levels of *Hk* and *Pgd* were increased by RE and further increased in the CT with the order HIIE‐RE. We supposed that the glycolytic ability was improved by our CT protocol.

Oxidative metabolism was evaluated using the expression levels of mitochondrial‐related genes and proteins in this study. CT enhanced oxidative metabolism, but without difference by order. PGC‐1α is a master regulator of mitochondrial biosynthesis (Hashimoto et al., 2007), and in human studies, they reported that gene expression levels of *Pgc*‐*1α* increase by endurance exercise, intensity‐dependent manner (Egan et al., 2016). Furthermore, PGC‐1α was also increased by resistance training (Ogasawara et al., 2016). The exercise intensity of CT in this study was the same between the groups, and it might be a matter of course that we did not detect the difference by order of CT. In addition, PGC‐1α and mitochondrial respiratory chain complex protein were significantly increased in CT groups, and oxidative metabolism was improved. The exercise protocol used in this study was appropriate, as HIIE improved glycolytic and oxidative metabolism in previous studies on humans (Perry et al., 2008). However, in this study, we could not measure swimming exhaustion time in performance tests, lipid oxidation, and citrate synthase activity. Therefore, in future research, the metabolic performance of the oxidative system should be evaluated.

## 5 CONCLUSION

In this study, we performed concurrent training combining RE and HIIE and examined the expression dynamics of mTOR signaling and signaling molecules of glycolytic metabolism. As a result, it was suggested that the expression of the signaling molecules was much enhanced by high intensity interval exercise first.

## CONFLICT OF INTEREST

The authors have no conflicts of interest to disclose.

## AUTHOR CONTRIBUTIONS

TS and TT conceived and designed the project; TS, HH, and KU performed the experiments; TS analyzed the data; TS wrote the manuscript and revisions; and the manuscript and revisions were checked by TT. All authors read and approved the final manuscript.
